# Unusual Maxillary Swelling in a 21-Year-Old Male – A Case Report

**DOI:** 10.30699/ijp.2025.2024697.3271

**Published:** 2025-01-10

**Authors:** Pushpak U Shah, Punnya. V. Angadi, Sanjay S Rao

**Affiliations:** 1 *Department of Oral Pathology and Microbiology, KLE VK Institute of Dental Sciences, KLE Academy of Higher Education and Research (KAHER), Karnataka, India*; 2 *Department of Oral and Maxillofacial Surgery, KLE VK Institute of Dental Sciences, KLE Academy of Higher Education and Research (KAHER), Karnataka, India*

**Keywords:** Ewing, Head and Neck, Maxilla, Oral Cavity, Sarcoma

## Abstract

Ewing sarcoma (ES) of the head and Neck is a rare entity. The most common location for ES is long bones accounting for 58%, with head and neck accounting only for 3% of all the sites. Here we highlight a unique presentation of ES involving the maxillary region in a 21-year-old male. Patient presented with right maxillary swelling for the last 2 months. Imaging studies reveal osteolytic lesion involving the right maxilla. Ewing's sarcoma rarely affects the head and neck region, posing diagnostic challenges as it mimics many common lesions. Accurate diagnosis requires a multidisciplinary approach involving clinical, radiological, histopathological, and molecular evaluations.

## Introduction

James Ewing, an American pathologist, first identified Ewing sarcoma (ES) in 1921. ES is the second most common bone malignancy in children (1), but it constitutes only 2% to 3% of cases in the head and neck region, which complicates its diagnosis due to overlapping clinical features with more common lesions. This case emphasizes the complexity of diagnosing Ewing sarcoma in atypical locations, highlighting the necessity for a multidisciplinary approach involving clinical, radiological, and histopathological evaluations to ensure accurate diagnosis and appropriate management.

## Case Report

**Fig. 1:**
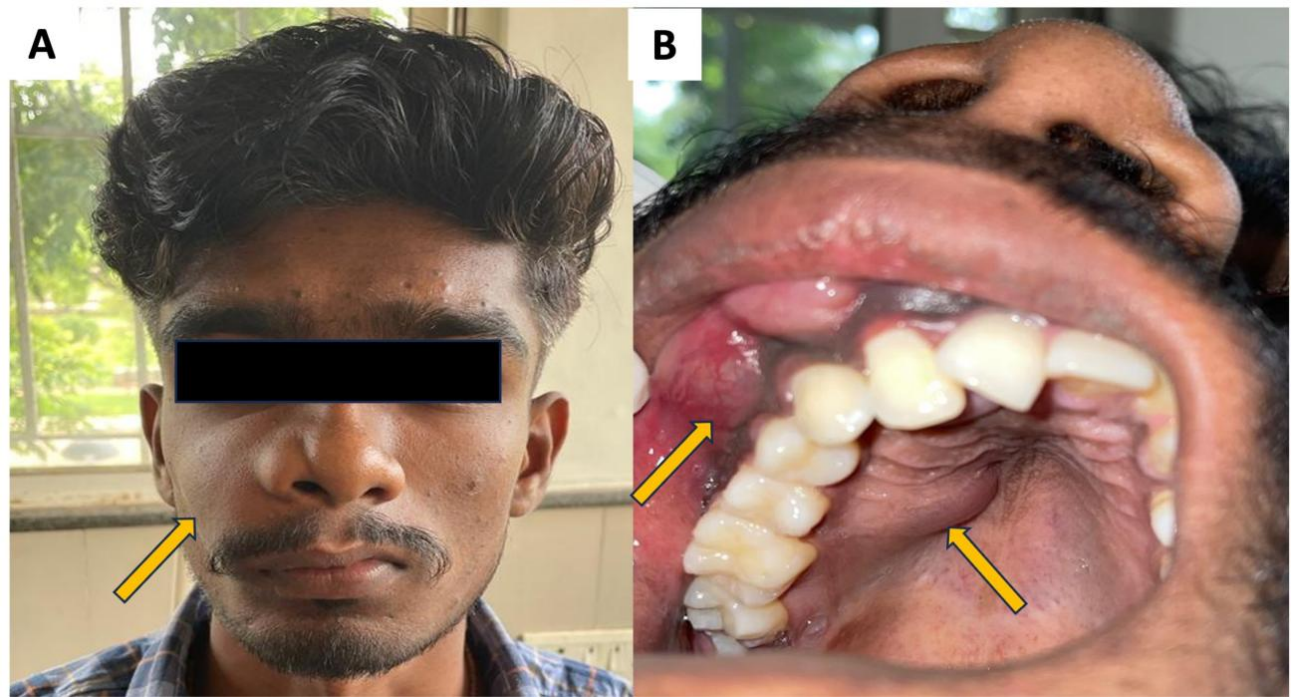
A: Extraoral swelling in the right maxillary region (yellow arrow). B: Intraoral swelling obliterating the buccal vestibule (arrow)and extending up to the mid-palatal region (arrow).

A 21-year-old male presented with a swelling in the right maxilla for 2 months. The swelling had slowly increased to its current size. Extraorally, the swelling measured approximately 4 × 5 cm, extending superoinferiorly from the infraorbital region to the angle of the mouth and anteroposteriorly from the midline of the nose to the line corresponding to the outer canthus of the eye. There was an elevation of the right nostril and obliteration of the nasolabial fold, causing facial asymmetry. The swelling was firm in consistency and non-tender on palpation (Figure 1).

Intraoral examination revealed effacement of the buccal vestibule and a nodular swelling extending from the right maxillary central incisor to the first molar, measuring 3 × 5 cm. The swelling appeared to extend on the palatal side along the midline and posteriorly to the right. It appeared soft in consistency and was non-tender to palpation. Displacement of the right maxillary central incisor (11) left maxillary central incisor (21), and lateral incisor (12) was observed. Mobility was noted in the maxillary right central and lateral incisors (11, 12), canine (13), second premolar (15), and first molar (16). A single right submandibular lymph node was palpable and non-tender, suggesting cortical plate perforation without ulceration. A clinical provisional diagnosis of ameloblastoma, odontogenic myxoma, or odontogenic keratocyst was considered.

### Differential Diagnosis

Ameloblastoma is an odontogenic tumor of epithelial origin, usually seen in tooth-bearing areas, with the mandible—followed by the maxilla—being a common site of occurrence. Maxillary tumors, though more common in the molar/ramus area, can occasionally be seen in the anterior regions involving the maxillary sinus and the nasal cavities. It is a slow-growing but locally invasive neoplasm, often mistaken for a low-grade malignancy, with evidence of expansion of cortical plates, displacement of teeth, and resorption of roots, as demonstrated in our case.[1]

Odontogenic Keratocyst is a distinct odontogenic cyst of developmental origin arising from the remnants of the dental lamina. It can occur anywhere within the jaws, with approximately 75% appearing in the posterior mandible, followed by the posterior maxilla and the maxillary sinus region. Most cases occur in individuals 10 to 40 years of age and exhibit aggressive clinical behavior, including expansion of cortical plates, displacement of teeth, and root resorption (2).

Odontogenic Myxoma is a mesenchymal odontogenic tumor that exhibits rapid growth and infiltrates the surrounding tissues. The posterior mandible is the most common site; however, involvement of the maxilla—where the odontogenic myxoma can fill the maxillary antrum—has also been frequently reported. The clinical presentation includes expansion of cortical plates, displacement of teeth, and root resorption, as seen in the present case (1).

### Radiological Findings

**Fig. 2:**
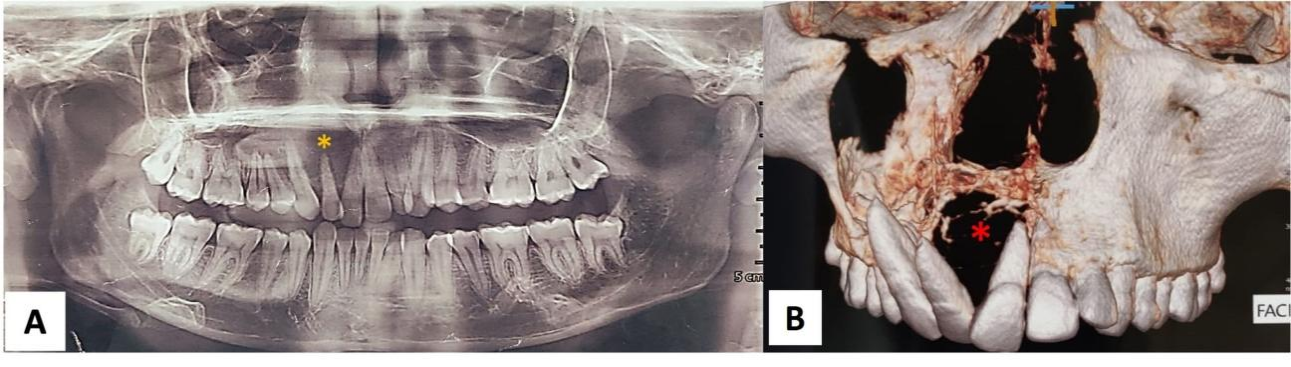
A: OPG reveals a radiolucent lesion (yellow asterisk) with displacement of the maxillary anterior teeth. B: CBCT reveals osteolytic lesion with displacement of the maxillary anterior teeth (red asterisk)

An orthopantomograph (OPG) and Cone Beam Computed Tomography (CBCT) were advised. The OPG revealed an ill-defined radiolucency extending from the right maxillary central incisor to the first molar, with displacement of 11, 12, 13, and 15, and root resorption of the mesiobuccal root of 16 (Figure 2A).

A CBCT scan was obtained with 0 mm thin slices at 1 mm intervals, revealing a large, unilocular radiolucency on the right side of the maxillary bone, extending from the 18 to 21 region. The lesion involved the right maxillary sinus with complete opacification of the sinus cavity. Superiorly, an expanded radiopaque border (elevated periosteum) was noted within the sinus cavity, contacting the sinus roof/orbital floor. The right orbital floor appeared continuous but thin and did not appear elevated.

There was a complete loss of alveolar bone in the 11, 12, 13, and 14 regions. The lesion extended medially to involve the palatine process of the maxilla in the 11, 12, 13, 14, and 15 regions, showing expansion and thinning of the inferior surface of the palatal process and thinning of the superior surface/nasal floor. The severely expanded anterolateral border of the lesion appeared scalloped and showed thin, wispy septae anteriorly. Internally, the lesion appeared homogeneously radiolucent (soft tissue/fluid radiodensity). The lesion’s maximum approximate dimensions were 40.7 mm (height) × 62.6 mm (anteroposteriorly) × 40.0 mm (mediolaterally) (Figure 2B). The radiological differential diagnosis included ameloblastoma, odontogenic keratocyst, and odontogenic myxoma.

### Histopathological Findings

**Fig. 3:**
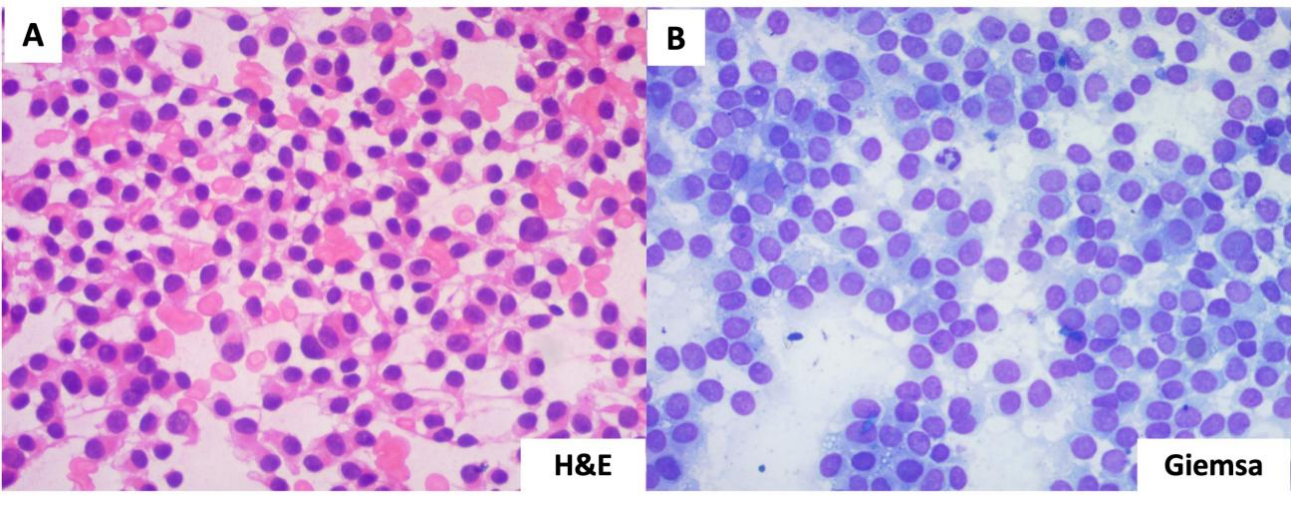
A & B: Cytological smears shows uniform round to ovoid cells with minimal cytoplasm suggestive of a round cell neoplasm (A: H& E 40x, B: Giemsa 40x)

Fine Needle Aspiration Cytology (FNAC) was performed, revealing an abundant amount of blood-tinged fluid. The hematoxylin and eosin (H&E)- and Giemsa-stained smear showed numerous round to ovoid cells with sparse eosinophilic cytoplasm, arranged in sheets and nests in a hemorrhagic background (Figure 3 A & B). A provisional diagnosis of a round cell neoplasm was considered.

An incisional biopsy was performed with an intraoral approach, and two brownish-white, soft tissue samples measuring 1.2 × 0.8 cm and 1.3 × 1.2 cm were received.

**Fig. 4:**
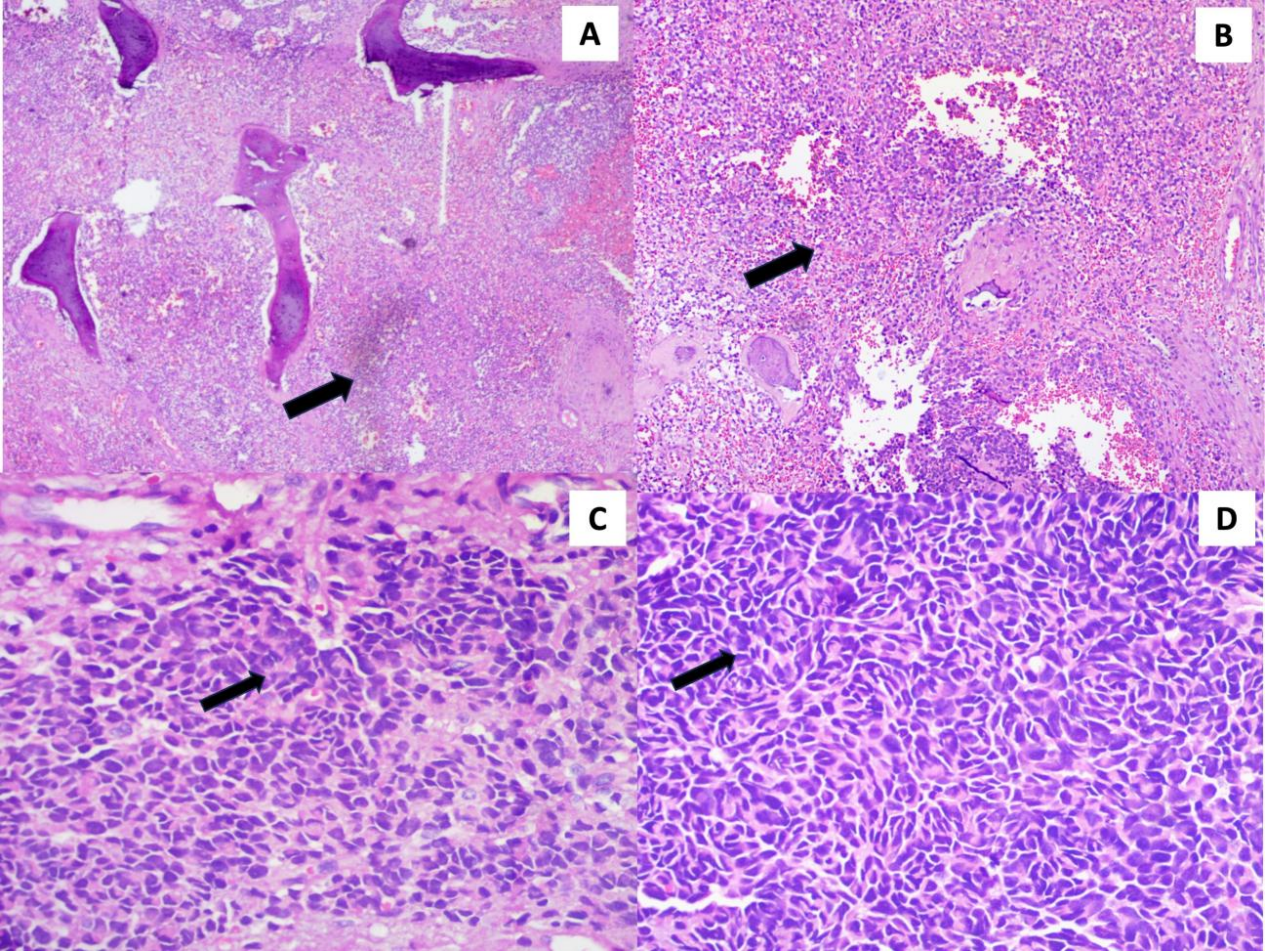
A & B: Photomicrographs show dense fibro cellular stroma with aggregates of hyperchromatic tumor cells interspersed in the stroma invading and destroying the bone (H&E,4x, 10x). C&D: Monomorphic proliferation of small round blue cells with well-defined hyperchromatic nuclei and scant cytoplasm (haematoxylin and eosin, 40x).

Under scanner view, the H&E-stained section showed multiple pieces of tissue consisting of dense sheets of darkly stained round to ovoid cells replacing the fibrocellular connective tissue and extending into muscle and bone (Figure 4 A & B).

Under higher magnification, the section showed round to ovoid cells with closed-faced, condensed nuclei arranged in sheets and aggregates. The cells, having minimal cytoplasm, appeared granular and focally exhibited eccentrically placed nuclei. A few sheets of cells displayed vesicular nuclei with scanty cytoplasm. Most of the cells were monomorphic, with minimal cytologic atypia, and mitosis was very sparse. These cells were separated by minimal stromal connective tissue and remnants of bone. Areas of hemorrhage were observed, with varying amounts of vascularity and dilated blood vessels. Some areas of cystic degeneration and island formation were also noted (Figure 4 C & D).

**Fig. 5:**
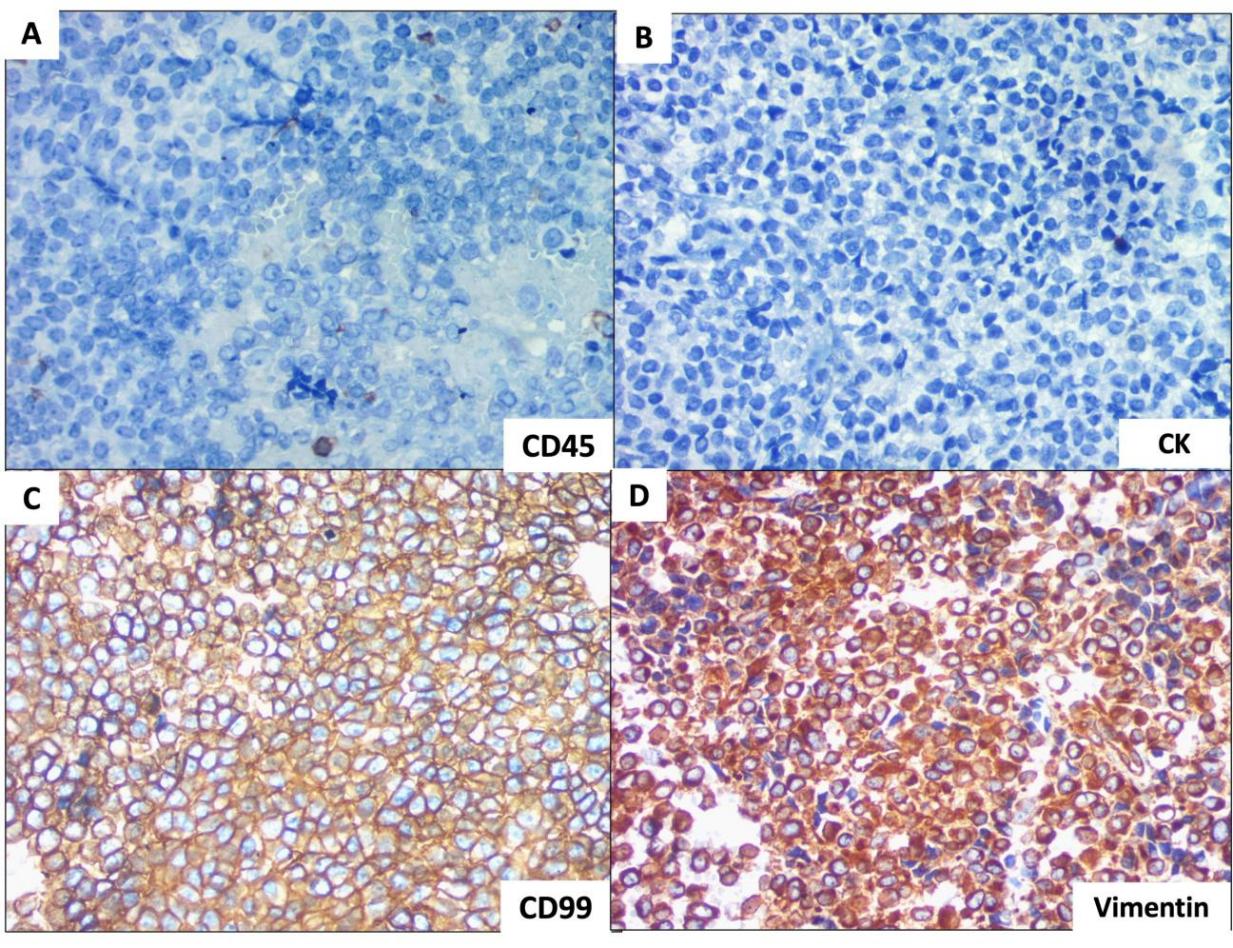
The round cells on immunohistochemistry were negative for CK and CD45 (A&B,40x) while positive for Vimentin and CD99(C&D, 40x)

The tumor cells were periodic acid–Schiff (PAS) positive. Other potential diagnoses for round-cell malignancy included Ewing’s sarcoma/PNET, neuroblastoma, mesenchymal chondrosarcoma, and lymphoma. Immunohistochemistry was performed for round-cell tumors using vimentin, CD99, NSE, CD45, and cytokeratin. The tumor cells were positive for CD99 and vimentin (Figure 5 C & D) but negative for CK and CD45 (Figure 5 A & B). A final diagnosis of Ewing sarcoma (ES) was thus confirmed.

A PET scan was performed before surgery to evaluate metastasis, revealing a heterogeneous, low-grade metabolically active osteolytic lesion in the right maxilla and maxillary sinus. A few low-grade metabolically active right cervical lymph nodes were noted, and a few ametabolic bilateral lung nodules were also seen. The patient was referred to a higher center for further evaluation and treatment and has since been lost to follow-up.

## Discussion

James Ewing first described Ewing sarcoma in 1921. He called it a “diffuse endothelioma of bone” because he believed the tumor originated from the undifferentiated vascular component of bone (2). Other names proposed included periothelioma, endothelial myeloma, reticuloendothelioma, reticular sarcoma, and intramedullary sarcoma. Ewing sarcoma is the second most frequently occurring bone malignancy among children and adolescents (3). In 1928, Oberling coined the term “Ewing Sarcoma” (4). The World Health Organization designated it as “Ewing Sarcoma/primitive neuroectodermal tumor,” based on the hypothesis that both entities belong to the same process and share the same genetic alteration, i.e., translocation 11:22.

Ewing sarcoma is considered a translocation sarcoma, being one of the first cancers identified pathologically as a unique entity characterized by a cytogenetic translocation. Chromosomal translocations in Ewing sarcoma involve the EWS (EWSR1) gene at 22q12 and a gene from the ETS family of transcription factors. This translocation creates the fusion gene EWS-FLI1 in about 90% of cases. The fusion gene produces a chimeric protein combining the N-terminal portion of EWS with the C-terminal portion of FLI1 (which contains the ETS DNA-binding domain). This abnormal protein acts as an aberrant transcription factor that drives cancer development. In the remaining cases, a different fusion gene, EWS-ERG, forms, where the ERG gene from 21q22 replaces FLI1 from 11q24. EWS-ETS fusions are hypothesized to initiate oncogenesis in Ewing sarcoma by promoting proliferative events.

Ewing sarcoma typically presents as a “small round blue cell tumor” composed of undifferentiated cells that offer minimal clues regarding their origin—distinct from sarcomas like osteosarcoma and liposarcoma, which exhibit clearer differentiation pointing to their specific lineages. The etiopathogenesis of Ewing sarcoma is unknown but is hypothesized to involve stem cells, neuroectodermal cells, immature reticular cells, or progenitor mesenchymal cells. Recent electron microscopic and immunohistochemical studies suggest a neurogenic origin (5).

Ewing sarcoma is the second most commonly occurring bone tumor after osteosarcoma in childhood (4). It is a primary malignant bone tumor accounting for 4–6% of cases, mainly affecting the long bones. It predominantly occurs in children and young adults aged 5–20, with a male predilection (3).

However, some authors report an average age of 36 years with no gender predilection (5). Head and neck bones are affected in only 2–3% of cases, with the most common sites being the mandibular region and skull base, followed by the orbit, nasal passages, and paranasal sinuses (6). In the present case, a 21-year-old male presented with maxillary swelling and facial disfigurement, along with tooth mobility, leading to a provisional diagnosis of an odontogenic cyst/tumor. Several authors have likewise misdiagnosed similar presentations as odontogenic cysts or tumors (8). An acute inflammatory response following trauma was also considered a provisional diagnosis (3,7)

The histopathological features of Ewing sarcoma and ES/pPNET are similar to other small round blue cell tumors, such as neuroblastoma, lymphoma, and rhabdomyosarcoma (5,9). These tumors comprise monomorphic small blue cells, which was also noted in the present case. Casaroto et al. believed that ES and ES/pPNET are two different diseases with diverse origins and sites, representing two ends of a spectrum with varied histopathological differentiation (3).

Diagnosis is based on clinical, histological, and immunohistochemical parameters. ES and ES/pPNET are analyzed for CD99, vimentin, and PAS. Schultze-Mosgau noted that CD99 expression in Ewing sarcoma is not entirely unique, but CD99 testing remains mandatory because 95% of ES cases are positive for this marker (3,10). CD99 is expressed on the cell surface (8). In our case, the tumor was positive for vimentin and CD99 and negative for CK, NSE, and CD45. Next-generation sequencing (NGS) has become an essential tool for diagnosing small round cell tumors in combination with routine histopathology and immunohistochemical findings, having identified previously undetected ES fusion transcripts that routine FISH or RT-PCR analyses missed.

### Treatment

Chemotherapy has raised the 5-year survival rate for localized disease from 10% to 70%. The standard treatment involves 12 weeks of neoadjuvant multi-agent chemotherapy, followed by surgery, radiotherapy, or both to manage and eliminate the disease, significantly improving outcomes.

Ewing sarcoma (ES) is radiosensitive, but advancements in surgical techniques have broadened its treatment options beyond expandable bones. Although radiotherapy is initially less complicated, it poses serious long-term risks such as growth retardation, fractures, and a high risk of subsequent primary neoplasms (SPN). Therefore, surgical resection with a wide margin is now preferred for local treatment. The choice between surgery and radiotherapy is most often debated when the primary tumor is located in the extremities (13).

### Prognosis

For Ewing's sarcoma, the most unfavorable prognostic factor is the presence of distant metastasis at diagnosis. Patients with metastases have only about a 20% chance of long-term survival, even with aggressive treatment. Those with bone or bone marrow metastasis at diagnosis have a poorer prognosis compared to patients with isolated pulmonary metastases.

Additional negative prognostic factors include being over 10 years of age, having a tumor larger than 200 mL, involvement of more central lesions (such as the pelvis or spine), and a poor response to chemotherapy. Histological grade is not prognostically significant in Ewing's sarcoma, as all cases are considered high-grade. However, indicators such as fever, anemia, elevated levels of white blood cells (WBC), erythrocyte sedimentation rate (ESR), and lactate dehydrogenase (LDH) suggest more extensive disease and a poorer prognosis.

Recently, the EWS/Fli1 fusion transcript has emerged as a prognostically relevant factor, with patients showing improved disease-free survival compared to those with other fusion transcript types. Nevertheless, some authors have found no significant clinical differences between tumors with EWS/Fli1 and EWS/ERG fusion transcripts (14).

## Conclusion

Ewing’s sarcoma is an osteolytic lesion primarily affecting the long bones and rarely involves the head and neck region. Diagnosing Ewing’s sarcoma in the head and neck poses significant challenges due to its clinical presentation, which often mimics more commonly occurring lesions. Accurate diagnosis necessitates a multidisciplinary approach, incorporating clinical, radiological, histopathological, and molecular evaluations to ensure appropriate management. The management involves chemotherapy, surgery, and radiotherapy. Recent advances in the treatment protocol have significantly affected morbidity and mortality.
